# Levosimendan Efficacy and Safety: 20 Years of SIMDAX in Clinical Use

**DOI:** 10.1097/FJC.0000000000000859

**Published:** 2020-07-06

**Authors:** Zoltán Papp, Piergiuseppe Agostoni, Julian Alvarez, Dominique Bettex, Stefan Bouchez, Dulce Brito, Vladimir Černý, Josep Comin-Colet, Marisa G. Crespo-Leiro, Juan F. Delgado, István Édes, Alexander A. Eremenko, Dimitrios Farmakis, Francesco Fedele, Cândida Fonseca, Sonja Fruhwald, Massimo Girardis, Fabio Guarracino, Veli-Pekka Harjola, Matthias Heringlake, Antoine Herpain, Leo M. A. Heunks, Tryggve Husebye, Višnja Ivancan, Kristjan Karason, Sundeep Kaul, Matti Kivikko, Janek Kubica, Josep Masip, Simon Matskeplishvili, Alexandre Mebazaa, Markku S. Nieminen, Fabrizio Oliva, Julius G. Papp, John Parissis, Alexander Parkhomenko, Pentti Põder, Gerhard Pölzl, Alexander Reinecke, Sven-Erik Ricksten, Hynek Riha, Alain Rudiger, Toni Sarapohja, Robert H. G. Schwinger, Wolfgang Toller, Luigi Tritapepe, Carsten Tschöpe, Gerhard Wikström, Dirk von Lewinski, Bojan Vrtovec, Piero Pollesello

**Affiliations:** 1 Department of Cardiology, Faculty of Medicine, University of Debrecen, Debrecen, Hungary; 2 Department of Clinical Sciences and Community Health, Centro Cardiologico Monzino, IRCCS, Milan, Italy; 3 Department of Surgery, School of Medicine, University of Santiago de Compostela, Santiago de Compostela, Spain; 4 Institute of Anaesthesiology, University Hospital of Zurich, Zurich, Switzerland; 5 Department of Anaesthesiology, University Hospital, Ghent, Belgium; 6 Cardiology Department, Centro Hospitalar Universitario Lisboa Norte, CCUI, Faculdade de Medicina, Universidade de Lisboa, Lisbon, Portugal; 7 Department of Anaesthesiology, Perioperative Medicine and Intensive Care, Masaryk Hospital, J.E. Purkinje University, Usti nad Labem, Czech Republic; 8 Heart Diseases Institute, Hospital Universitari de Bellvitge, Barcelona, Spain; 9 Complexo Hospitalario Universitario A Coruña (CHUAC), CIBERCV, Instituto de Investigacion Biomedica A Coruña (INIBIC), Universidad de a Coruña (UDC), La Coruña, Spain; 10 Heart Failure and Transplant Program, Cardiology Department, University Hospital 12 Octubre, Madrid, Spain; 11 Department of Cardiac Intensive Care, Petrovskii National Research Centre of Surgery, Sechenov University, Moscow, Russia; 12 Department of Cardiology, Medical School, University of Cyprus, Nicosia, Cyprus; 13 Department of Cardiovascular, Respiratory, Nephrology, Anaesthesiology and Geriatric Sciences, La Sapienza University of Rome, Rome, Italy; 14 Heart Failure Clinic, São Francisco Xavier Hospital, CHLO, Lisbon, Portugal; 15 Department of Anaesthesiology and Intensive Care Medicine, Division of Anaesthesiology for Cardiovascular Surgery and Intensive Care Medicine, Medical University of Graz, Graz, Austria; 16 Struttura Complessa di Anestesia 1, Policlinico di Modena, Modena, Italy; 17 Dipartimento di Anestesia e Terapie Intensive, Azienda Ospedaliero-Universitaria Pisana, Pisa, Italy; 18 Emergency Medicine, Meilahti Central University Hospital, University of Helsinki, Helsinki, Finland; 19 Department of Anaesthesiology and Intensive Care Medicine, University of Lübeck, Lübeck, Germany; 20 Department of Intensive Care, Hôpital Erasme, Brussels, Belgium; 21 Department of Intensive Care Medicine, Amsterdam UMC, Amsterdam, the Netherlands; 22 Department of Cardiology, Oslo University Hospital Ullevaal, Oslo, Norway; 23 Department of Anaesthesiology, Reanimatology and Intensive Care, University Hospital Centre, Zagreb, Croatia; 24 Departments of Cardiology and Transplantation, Sahlgrenska University Hospital, Gothenburg, Sweden; 25 Intensive Care Unit, National Health Service, Leeds, United Kingdom; 26 Global Medical Affairs, R&D, Orion Pharma, Espoo, Finland; 27 Department of Cardiology and Internal Medicine, Nicolaus Copernicus University, Torun, Poland; 28 Intensive Care Department, Consorci Sanitari Integral, University of Barcelona, Barcelona, Spain; 29 Lomonosov Moscow State University Medical Centre, Moscow, Russia; 30 Department of Anaesthesiology and Critical Care Medicine, AP-HP, Saint Louis and Lariboisière University Hospitals, Paris, France; 31 Sydäntutkimussäätiö, Helsinki, Finland; 32 Department of Cardiology, Niguarda Ca'Granda Hospital, Milan, Italy; 33 MTA-SZTE Research Group of Cardiovascular Pharmacology, Hungarian Academy of Sciences, University of Szeged, Szeged, Hungary; 34 Second Department of Cardiology, Attikon University Hospital, National and Kapodistrian University of Athens, Athens, Greece; 35 Emergency Cardiology Department, National Scientific Centre MD Strazhesko Institute of Cardiology, Kiev, Ukraine; 36 Department of Cardiology, North Estonia Medical Centre, Tallinn, Estonia; 37 Department of Internal Medicine III, Cardiology and Angiology, Medical University of Innsbruck, Innsbruck, Austria; 38 Klinik für Innere Medizin III, Kardiologie, Universitätsklinikum Schleswig-Holstein, Kiel, Germany; 39 Department of Anaesthesiology and Intensive Care, Sahlgrenska University Hospital, Gothenburg, Sweden; 40 Department of Anaesthesiology and Intensive Care Medicine, Cardiothoracic Anaesthesiology and Intensive Care, Institute for Clinical and Experimental Medicine, Prague, Czech Republic; 41 Department of Medicine, Spittal Limmattal, Schlieren, Switzerland; 42 Statistical Services, R&D, Orion Pharma, Espoo, Finland; 43 Medizinische Klinik II, Klinikum Weiden, Teaching Hospital of University of Regensburg, Weiden, Germany; 44 Department of Anaesthesiology and Intensive Care Medicine, Medical University of Graz, Graz, Austria; 45 Anaesthesia and Intensive Care Division, San Camillo-Forlanini Hospital, Rome, Italy; 46 Department of Cardiology, Campus Virchow Klinikum, Charité—University Medicine Berlin, Berlin, Germany; 47 Institute of Medical Sciences, Uppsala University, Uppsala, Sweden; 48 Department of Cardiology, Myokardiale Energetik und Metabolismus Research Unit, Medical University of Graz, Graz, Austria; 49 Department of Cardiology, Advanced Heart Failure and Transplantation Centre, University Clinical Centre, Ljubljana, Slovenia; 50 Critical Care Proprietary Products, Orion Pharma, Espoo, Finland.

**Keywords:** acute heart failure, advanced heart failure, hemodynamics, inodilator, inotrope, neurohormone, regulatory clinical trial

## Abstract

Levosimendan was first approved for clinical use in 2000, when authorization was granted by Swedish regulatory authorities for the hemodynamic stabilization of patients with acutely decompensated chronic heart failure (HF). In the ensuing 20 years, this distinctive inodilator, which enhances cardiac contractility through calcium sensitization and promotes vasodilatation through the opening of adenosine triphosphate–dependent potassium channels on vascular smooth muscle cells, has been approved in more than 60 jurisdictions, including most of the countries of the European Union and Latin America. Areas of clinical application have expanded considerably and now include cardiogenic shock, takotsubo cardiomyopathy, advanced HF, right ventricular failure, pulmonary hypertension, cardiac surgery, critical care, and emergency medicine. Levosimendan is currently in active clinical evaluation in the United States. Levosimendan in IV formulation is being used as a research tool in the exploration of a wide range of cardiac and noncardiac disease states. A levosimendan oral form is at present under evaluation in the management of amyotrophic lateral sclerosis. To mark the 20 years since the advent of levosimendan in clinical use, 51 experts from 23 European countries (Austria, Belgium, Croatia, Cyprus, Czech Republic, Estonia, Finland, France, Germany, Greece, Hungary, Italy, the Netherlands, Norway, Poland, Portugal, Russia, Slovenia, Spain, Sweden, Switzerland, the United Kingdom, and Ukraine) contributed to this essay, which evaluates one of the relatively few drugs to have been successfully introduced into the acute HF arena in recent times and charts a possible development trajectory for the next 20 years.

## Abstract 2

Supplemental Digital Content is Available in the Text.

## ORIGINS OF A UNIQUE CARDIOVASCULAR AGENT

Before the 1980s, therapy to enhance cardiac contractility in heart failure (HF) substantially meant oral digitalis glycosides, supplemented by beta-adrenergic agonists such as dopamine or dobutamine (introduced in the middle of the 1970s) in acute situations.^1^ It was therefore a matter of some note when the US Food and Drug Administration approved a new agent as a short-term IV therapy for patients with refractory HF. Amrinone was the product of a widespread research initiative that recognized the limitations of existing inotropic therapy and that, equipped with a new understanding of the cellular mechanisms of cardiac contractility, set out to develop what respected commentators of the time referred to as “non-glycoside, non-sympathomimetic positive inotropic agents.”^2–4^ Amrinone was the first agent to reach clinical use from the small but important family of phosphodiesterase (PDE) inhibitors, which would later include milrinone and enoximone.^5,6^ However, despite being nonsympathomimetic positive inotropic agents, all PDE inhibitors, in common with the catecholamines, were shown to be calcium mobilizers, probably due to their limited selectivity toward specific key PDE isoforms, and shared with catecholamines some unwanted effects intrinsic to any drug that raises intracellular calcium. In fact, all calcium mobilizers, by definition, exert an inotropic effect by providing increased ionic calcium levels for the contractile protein machinery, a process that may ultimately prove detrimental to individual cardiomyocytes and therefore also to patients.^7^

At about the same time, a new concept was proposed by the independent groups of J Caspar Rüegg in Heidelberg and R. John Solaro in Chicago, namely the potential of new agents to enhance the sensitivity to calcium of key targets in the contractile apparatus instead of increasing the intracellular calcium transient to augment contractility.^8,9^ In 1984, Rüegg et al^10^ described the pharmacology of a new agent, later known as pimobendan, which combined PDE inhibitor activity with a direct calcium-sensitizing effect.

It was in this climate of innovation that the new chemical entity R-((4-(1,4,5,6-tetrahydro-4-methyl-6-oxo-3-pyridazinyl)phenyl)hydrazono)propanedinitrile, known by the identifier OR-1259 at the time, appeared in the published records. An abstract was published in 1992 describing “a positive inotropic and vasodilatory compound with antiarrhythmic properties.”^11^ This preliminary report noted that OR-1259 exerted a positive inotropic effect despite a reduction in the voltage-sensitive Ca^2+^ current. As is not uncommon in abstract reports, the authors advised that “Further studies… are in progress.”

In 1995, Heimo Haikala reported the findings of in-depth research into the mechanism of action of this agent in his pioneering paper.^12^ At the same time, an article describing the binding of a new Ca^2+^ sensitizer, levosimendan, to recombinant human cardiac troponin C was also published.^13^ Those first descriptions may be regarded as foundation publications in the chronology of this drug and a starting point for the PubMed-cited literature on levosimendan, which had expanded to almost 1500 reports by the end of 2019.

Levosimendan was described as “a calcium sensitiser rationally designed and screened to act through its calcium-dependent binding to cardiac troponin C,” and the experimental basis for this description was set out in detail.^12^ From the beginning, clear mechanistic differences were spotted between levosimendan and several other drugs then in development, including pimobendan, MCI-154, and EMD 53998. Levosimendan was a first-in-class agent at the time of its emergence, promoting inotropy mainly through calcium sensitization of cardiac troponin C (cTnC). More than 20 years later it remains, remarkably, an only-in-class drug, with a mechanism of action that clearly differentiates it from adrenergic agents.

**FIGURE 1.**
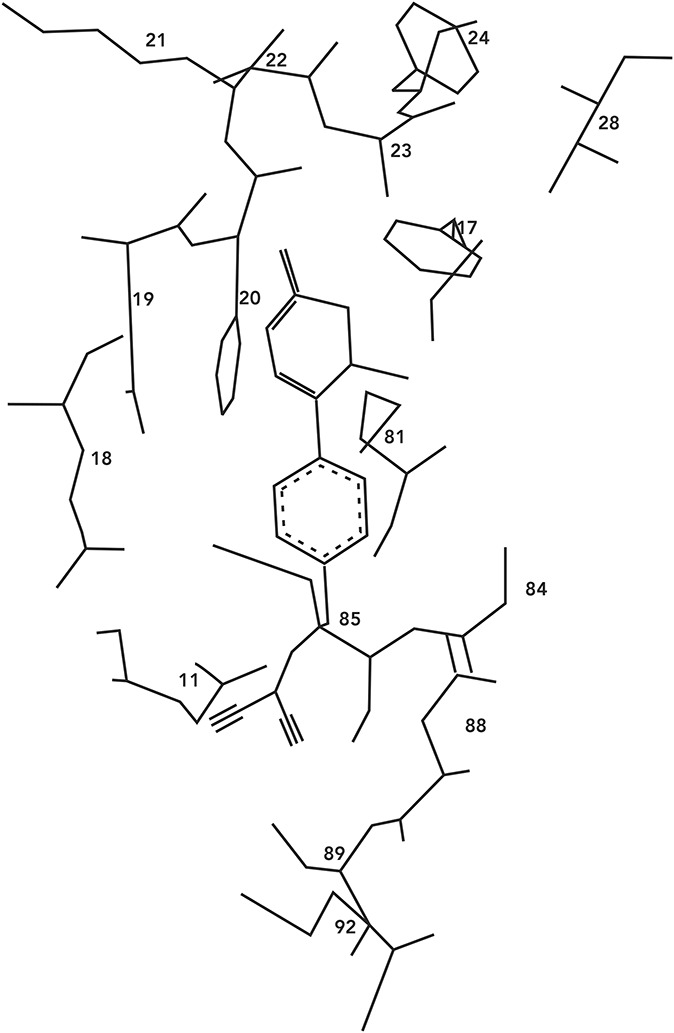
Early molecular model of the levosimendan–cTnC complex: The dihydropyridazinone ring of levosimendan is enclosed within a hydrophobic cleft formed by the amino acid residues Phe^20^, Ala^22^, Ala^23^, Phe^24^, Val^28^, and Phe^77^, and the phenyl ring of levosimendan is aligned to Met^81^, Cys^84^, and Met^85^. Source: Pollesello et al^13^ Reproduced with permission from the American Society for Biochemistry and Molecular Biology. cTnC = cardiac troponin C.

Levosimendan, as reported by Pollesello et al^13^ in 1994, binds to calcium-saturated human cTnC “in a hydrophobic patch of the N-domain near the site where the B helix is located when the protein is in its apoform.” Figure 1 shows an original diagram from the 1994 paper proposing a molecular model of the drug–ligand complex.

That interaction leads to a stabilization of the calcium-bound conformation of the regulatory (or N) domain of cTnC, which in turn causes a change in the conformation of the ‘switch’ region of cardiac troponin I (cTnI) and detachment of cTnI from actin filaments.^14^ Removal of the inhibitory effects of cTnI facilitates the formation of actin–myosin cross-bridges and the disinhibition of actomyosin adenosine triphosphate (ATP) synthase, resulting in enhanced cardiac contractility.^12,13,15–18^ These findings, confirming that the binding of levosimendan to TnC is linked to calcium sensitization, proved to be the first stage of what has since matured into a long-lasting research trail.^19–22^

The calcium-sensitizing action of levosimendan is manifested as a leftward shift in the curve describing the relation between contractile force and calcium concentration, achieved through a direct effect on cTnC. That augmentation of contractility is not associated with increases in calcium transients, intracellular calcium, or myocardial oxygen consumption and is not compromised by pretreatment with beta-blockers. It should also be noted that the interaction between levosimendan and cTnC was shown to be more intense at high, systolic ionic calcium levels than at low, diastolic calcium levels, thus avoiding impairment of myocardial relaxation upon levosimendan administration.

In addition to its principal action as a calcium-sensitizing agent, levosimendan was found in the course of its development program to mediate the opening of ATP-dependent potassium channels (K_ATP_ channels) in vascular smooth muscle cells in various vascular beds.^23^ By this mechanism of action, levosimendan induces an increase in blood perfusion in key organs and a systemic vasodilatation when levosimendan is used at doses within the recognized therapeutic range, which means that the drug must be considered and used as an inodilator and not simply as an inotrope. An essential aspect of the pharmacology and clinical profile of levosimendan is that its perfusion enhancement and systemic vasodilation effects are mediated through different mechanisms and may therefore be disentangled from each other. Levosimendan—acting on K_ATP_ channels—has a different regional/peripheral versus systemic effect when compared with drugs such as the PDE inhibitors.^24^

Separate emphasis must be placed on the discovery that levosimendan also opens the K_ATP_ channels on the mitochondrial inner membrane.^25,26^ This effect has been associated with cardioprotection, infarct size reduction, and mitigation of ischemia/reperfusion injuries in a range of in vitro, ex vivo, and in vivo studies in nonhuman species^27–31^ and in clinical studies.^32^

The aforementioned effects deriving from calcium sensitization and vasodilation are shared by the long-acting levosimendan metabolite OR-1896, which is formed in the intestine through a reduction–acetylation pathway.^33–35^ Free plasma concentrations of the parent drug and the metabolite are similar, but clinically meaningful plasma concentrations of pharmacologically active OR-1896 are detectable for days after an infusion of levosimendan and contribute to the persistence of the therapeutic effect after administration of the parent drug is stopped.^36^

**FIGURE 2.**
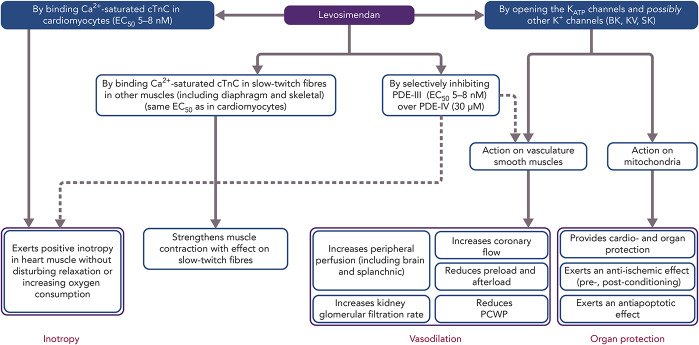
Mode of actions and pharmacologic effects of levosimendan: The mechanisms of action in the blue boxes contribute to the cardiovascular effects of the drug. Dotted lines mark pathways that are still not fully elucidated. EC_50_, half maximal effective concentration; K_ATP_, adenosine triphosphate–dependent potassium channels; PDE III, IV, phosphodiesterase isoforms in cardiac tissue. Adapted from: Al-Chalabi et al^216^ Used with permission from Wolters Kluwer Health.

Beyond these primary mechanisms, levosimendan has been identified as having a range of ancillary actions (often described as pleiotropic effects) that do not involve an enhancement of cardiac function but which may be implicated in some of the clinical effects of and responses to levosimendan.^37^ These include anti-inflammatory, antioxidative, and antiapoptotic actions that may be exerted in noncardiac organs, including the kidneys, liver, gut and splanchnic vasculature, lungs, and/or respiratory muscles (Fig. 2).

Levosimendan inhibits only one isoform of intracellular PDE enzymes (PDE-III) and in a highly selective manner. Of note, the PDE-III over PDE-IV isoform selectivity of levosimendan is the highest known to date, with a ratio of 10,000, compared with 14 for milrinone.^38,39^ It was proposed that inhibition of only the PDE-III isoform, not that of PDE-IV, would be insufficient to increase intracellular levels of cyclic adenosine monophosphate (cAMP) to the same levels as by simultaneous inhibition of the 2 isozymes.^38^ This lack of PDE dependence further differentiates levosimendan from nonselective PDE inhibitors, such as milrinone, and provides an explanation of their different pharmacological behaviors, as in the case of the oxygen consumption to force production ratios.^38,40–42^

However, this interpretation is not unanimous. Maack et al^43^ have proposed that PDE-III inhibition by levosimendan may indeed play a relevant role in the pharmacological effects of levosimendan. In that interpretation, PDE-III inhibition by levosimendan synergizes with Ca^2+^ sensitization for the resulting inotropic action. Interestingly, from this synergy, the authors predict that the more beta-adrenergic receptors are preactivated by endogenous or exogenous catecholamines, the more pronounced will be the inotropic effect of levosimendan, and the more this effect would be mediated by PDE-III inhibition rather than by Ca^2+^ sensitization. Conversely, at low preactivation of beta-adrenergic receptors (such as during pharmacological beta-blockade), the Ca^2+^-sensitization effect of levosimendan would become more important for inotropy. The take-home message of a consensus paper from the Translational Working Group of the Heart Failure Association of the European Society of Cardiology (ESC) is that long-term use of drugs that exclusively target adrenergic signaling (eg, catecholamines and PDE inhibitors) is associated with adverse outcomes, whereas levosimendan, with its hybrid calcium sensitization and PDE-III inhibition properties, should be given the benefit of the doubt and further attention.

The effect of levosimendan has also been studied in the presence of beta-blockers and/or inopressors. Xanthos et al^44^ reported that the combination of epinephrine, atenolol, and levosimendan, when given during cardiac arrest and resuscitation in a pig model, resulted in improved 48-hour survival and postresuscitation cardiac function. Concurrently, Lochner et al^45^ reported that the effects of levosimendan were not blunted by the presence of beta-blockers, as in the case of adrenergic inotropes.

**FIGURE 3.**
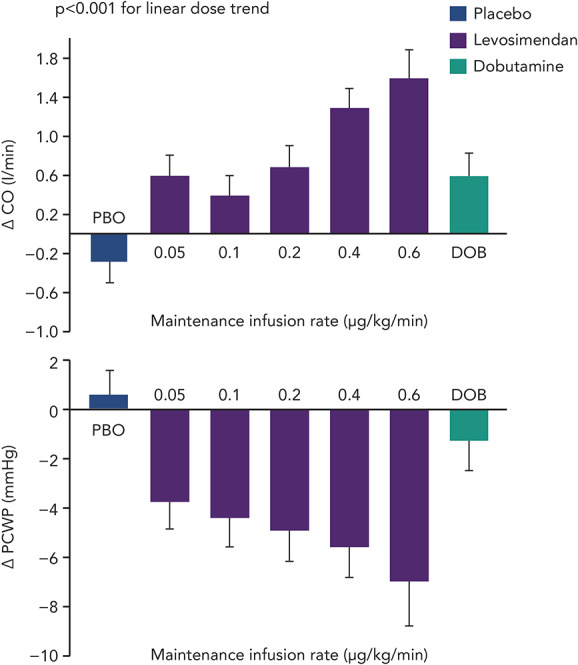
Change in CO and PCWP: Change from baseline at the conclusion of a 24-hour infusion of levosimendan (given as a 10-minute bolus of 6–24 µg/kg, then an infusion of 0.05–0.6 µg/kg/min), placebo, or dobutamine (6 µg/kg/min) in patients with stable HF. DOB, dobutamine; PBO, placebo. Data from: Nieminen et al.^52^

Levosimendan entered formal clinical evaluation and development in acute HF (AHF) in the mid-1990s.^46–48^ Initially, it was established that IV levosimendan produced dose-dependent increases in cardiac output (CO) and decreases in pulmonary capillary wedge pressure (PCWP; Fig. 3). Those effects were not accompanied by significant increases in myocardial energy consumption, thus confirming the paradigm envisioned on the basis of the preclinical data.^28,49–51^

## FROM BENCH TO BEDSIDE

Levosimendan entered clinical trials profiled as a novel inotrope with potential for the short-term treatment of acutely decompensated chronic HF. The regulatory studies program devised to evaluate it in this indication enrolled almost 4000 patients (Table 1) and produced the following key insights.

**TABLE 1.**
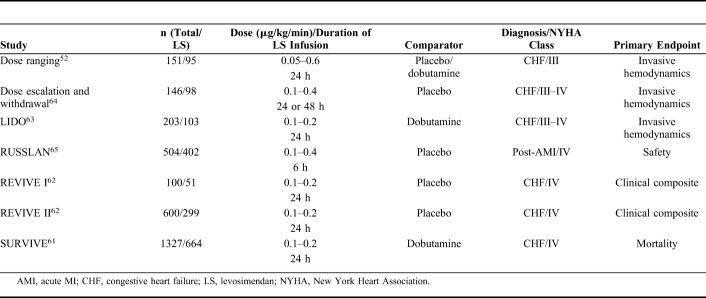
Regulatory Clinical Trials of Levosimendan

### Clinical Effects

#### Hemodynamic Effects

The hemodynamic effects of levosimendan seen in preclinical studies were confirmed. In patients with AHF, levosimendan achieves significant dose-dependent increases in CO and stroke volume and decreases in PCWP, mean blood pressure, mean pulmonary artery pressure, mean right atrial pressure, and total peripheral resistance.^52^

In line with preclinical data, clinical studies have confirmed that levosimendan does not have a negative effect on diastolic function. In contrast, levosimendan has lusitropic effects.^53,54^ Inodilation is not only seen in the left side of the heart; right ventricular contractility is also improved, and pulmonary vascular resistance is decreased.^55–57^

#### Pharmacokinetics in Clinical Trials

**FIGURE 4.**
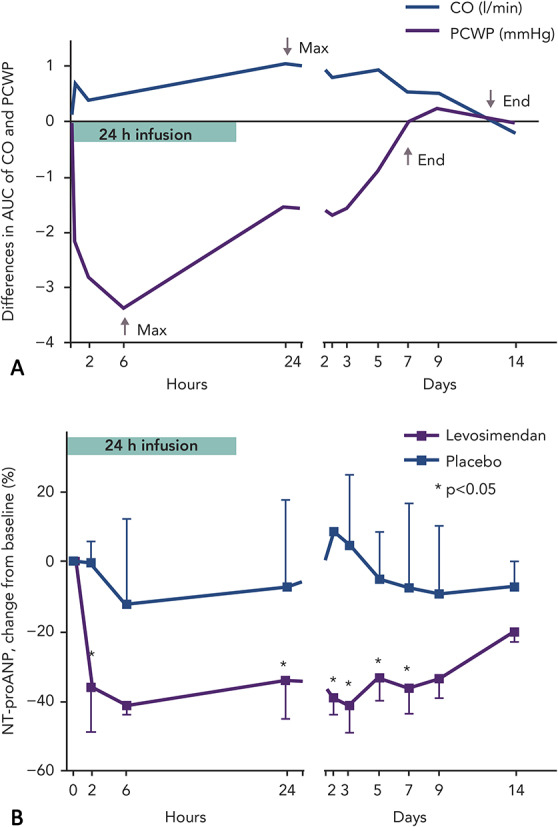
Pharmacokinetics of levosimendan: A, Differences in the area under the receiver operating characteristics curve (AUC) for changes in Doppler echocardiography–derived PCWP and CO in patients with acute HF treated with levosimendan or placebo (n = 11 in both groups) for 24 hours. Due to of the formation of the active metabolite, the hemodynamic effects are maintained several days after stopping levosimendan infusion. B, Median change in N-terminal prohormone atrial natriuretic peptide (NT-proANP) over 14 days in patients with HF receiving levosimendan or placebo (n = 11 in both groups) for 24 hours. Source: Lilleberg et al^58^ Reproduced with permission from John Wiley and Sons.

As anticipated in nonclinical studies, in humans the hemodynamic effects of a 24-hour infusion of levosimendan are protracted for several days in patients with AHF due to of the presence of an active metabolite (Fig. 4A).^58–60^

#### Effects on Neurohormones

Rapid and sustained reductions in levels of natriuretic peptides were characteristic of levosimendan in its regulatory clinical trials.^61–63^ The effect on natriuretic peptides closely follows the hemodynamic effects: both are evident for at least 1 week after the levosimendan infusion period (Fig. 4B).^58^ In the trial Survival of Patients with Acute Heart Failure in Need of IV Inotropic Support (SURVIVE), in patients with acute decompensated HF, changes in brain natriuretic peptide (BNP) levels up to 5 days after the start of infusion of levosimendan could be seen, which was not the case after 48 hours of treatment with dobutamine.^61^

#### Impact on Signs and Symptoms in AHF

Levosimendan induces a rapid and sustained improvement in symptoms, as evidenced by Packer et al^62^ and Slawsky et al.^64^ In the second of those studies, relief of dyspnea was reported in 29% of levosimendan-treated patients compared with 15% of the placebo-treated patients 6 hours after starting the infusion (*P* = 0.037).^64^ Improvement in symptoms was evident for up to 5 days.^62^ Data on the use of rescue medications in the Randomised Evaluation of IV Levosimendan Efficacy (REVIVE) program further confirm the effectiveness of levosimendan for symptom relief (Table 2).^62^ Dyspnea and fatigue symptoms also responded better to levosimendan than to dobutamine in the Levosimendan Infusion versus Dobutamine (LIDO) trial, although not to the level required for statistical significance.^63^

**TABLE 2.**
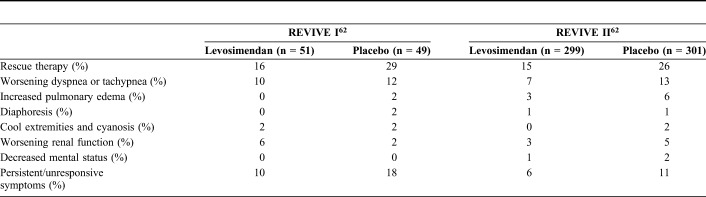
Use of Rescue Medications in the REVIVE Program

### Clinical Outcomes

#### Hospitalizations

Patients treated with levosimendan in the LIDO study spent significantly more days alive and out of hospital than dobutamine-treated patients in a retrospective 180-day follow-up analysis (median 157 vs. 133 days; *P* = 0.027).^63^ In the Randomised Study on Safety and Effectiveness of Levosimendan in Patients with Left Ventricular Failure After an Acute Myocardial Infarction (RUSSLAN), the combined risk of death and worsening HF was significantly lower in patients treated with levosimendan than in the control group during the infusion period (2% vs. 6%; *P* = 0.033) and at 24 hours (4% vs. 9%; *P* = 0.044).^65^ In the REVIVE II study, a greater percentage of patients treated with levosimendan than placebo were released within 5 days (46% vs. 37%), and the mean duration of the initial hospitalization was almost 2 days shorter (7.0 vs. 8.9 days).^62^ No significant intergroup difference was recorded in the SURVIVE trial (*P* = 0.3).^61^

#### Mortality

Thirty-one-day mortality in the LIDO trial indicated a survival advantage from levosimendan (mortality rate 8%, vs. 17% with dobutamine, HR 0.43, *P* = 0.049).^63^ This was corroborated in a retrospective extension of follow-up to 180 days (mortality rate 26%, vs. 38% with dobutamine, HR 0.57, *P* = 0.029). In RUSSLAN, a survival benefit from levosimendan persisted at 180-day follow-up (23% vs. 31%; *P* = 0.053).^65^

In the REVIVE and SURVIVE trials there were no significant differences in 3- and 6-month overall survival between the study groups.^61,62^ However, there was evidence of a survival gain from levosimendan treatment in SURVIVE patients who had a history of chronic decompensated HF or who were using beta-blockers.^66^ In patients with existing chronic HF (88% of the study population), mortality was lower in the levosimendan group than in the dobutamine group at day 5 [3.4% vs. 5.8%, HR 0.58, 95% confidence interval (CI) (0.33–1.01), *P* = 0.05] and at day 14 [7.0% vs. 10.3%, HR 0.67, 95% CI (0.45–0.99), *P* = 0.045]. In patients who used beta-blockers (50% of the study population), mortality was significantly lower for levosimendan than for dobutamine at day 5 [1.5% vs. 5.1%, HR 0.29, 95% CI (0.11–0.78), *P* = 0.01].

### Safety

A safety summary prepared by Orion Pharma in its capacity as sponsor of the regulatory studies found no difference in the proportion of patients with reduction in arterial blood pressure in response to treatment (23.1% vs. 23.1%), although REVIVE II, considered as a single study, diverged from this overall trend by showing more hypotension in the levosimendan arm.^62^ In 2012, Landoni et al^67^ collected data from 5480 patients in 45 randomized clinical trials and also carried out meta-analysis of the adverse events. No signals were seen for MI [data from 25 studies, RR 0.789, 95% CI (0.522–1.185), *P* = 0.3], ventricular arrhythmias [data from 9 studies, RR 0.885, 95% CI (0.611–1.281), *P* = 0.5], or supraventricular arrhythmias [data from 19 studies, RR 1.005, 95% CI (0.782–1.291), *P* = 0.9], but a numerical trend was seen for hypotension [data from 22 studies, RR 1.389, 95% CI (0.996–1.936), *P* = 0.53]. There are some contradictory or indirect and inconclusive reports related to the impact of levosimendan on platelet function, but a recent meta-analysis of 9 randomized controlled trials (RCTs) found that levosimendan did not increase postoperative bleeding risk.^68,69^ Moreover, in **Supplemental Digital Content 1** (see **Supplementary Appendix**, http://links.lww.com/JCVP/A472) of the large regulatory trial Levosimendan in Patients with Left Ventricular Systolic Dysfunction Undergoing Cardiac Surgery Requiring Cardiopulmonary Bypass (LEVO-CTS) no signs of increase in periprocedural or postprocedural hemorrhage were seen after treatment with levosimendan.^70^

Conversely, a later, independent meta-analysis of data from more than 5000 patients indicated increased risks of extrasystoles [RR 1.88, 95% CI (1.26–2.81)], hypotension [RR 1.33, 95% CI (1.15–1.53)], and headache or migraine [RR 1.94, 95% CI (1.54–2.43)] when compared with reference therapies.^71^ Retrospective analyses of the REVIVE II data set identified low blood pressure at baseline as a possible risk factor for the use of levosimendan, and the current, approved Summary of Product Characteristics reflects that finding.^72^

### Dosing

Levosimendan is given as a continuous infusion of 0.05 or 0.1 or 0.2 µg/kg/min for 24 hours, which may be preceded by a loading dose (bolus) of 6–12 µg/kg in 10 minutes. The loading dose was used in the active-controlled regulatory studies LIDO and SURVIVE, in which dobutamine served as comparator. Given that the elimination half-life of dobutamine is a few minutes while that of levosimendan is approximately 1 hour, the hemodynamic effects of dobutamine are seen almost immediately after the infusion is started, whereas a bolus of levosimendan is needed to see immediate effects. For consistency, all other studies in the regulatory clinical program were designed to include a bolus dose, followed by a maintenance infusion. It was later found that, in the case of hypovolemia or initial low blood pressure, a levosimendan bolus could be associated with hypotension or arrhythmias. Therefore, use of an initial bolus of levosimendan is now generally not recommended, and it has often been avoided in clinical practice and used only if an instant effect is sought and the systolic blood pressure is adequate.^73,74^

### Into Regular Clinical Use

The experience gained in regulatory studies provided the basis for the first approval of IV levosimendan, which was introduced in Sweden in 2000 for the management of AHF with the name SIMDAX. Since then, more than 60 jurisdictions have approved the drug, including most of the countries of the EU and Latin America. Levosimendan is currently in active clinical evaluation in the United States.

In the 20 years since its first introduction, IV levosimendan has been one of the notably few successful drugs entering the market in an underserved area of cardiovascular medicine: attempts at drug innovation in AHF have been characterized by repeated disappointments (either partial or total) or contradictory findings that have hindered progress.^75^

Levosimendan itself has not been immune to some of the frustrations of research in this area: in particular, the nonunivocal findings on 6-month mortality in its regulatory studies complicated the process of establishing its therapeutic niche. Innovation in this area may have been poorly served by a regulatory emphasis on longer-term survival effects. This was perhaps misaligned with clinical realities and led to an emphasis on large trials which, by aggregating data from patients with different underlying pathophysiologies plus variations in both pharmacological and nonpharmacological treatments, may have generated signal-to-noise ratios that precluded the identification of a meaningful effect on the central end point of all-cause mortality/survival. The unsuitability of all-cause long-term mortality as an index of therapeutic effect was acknowledged by experts in the field of HF about a decade ago, but that realization came too late to influence the conduct of the regulatory trials of levosimendan.^76,77^

**FIGURE 5.**
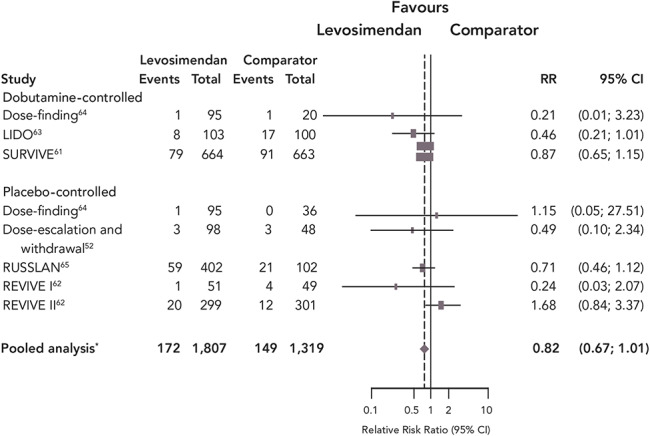
Effect of levosimendan on survival in the regulatory clinical trials: Meta-analysis of the clinical trials considered by regulatory authorities for the introduction of levosimendan. *Pooled statistic calculated using the Cochran–Mantel–Haenszel test, controlling for study. Source: Pollesello et al.^80^ Reproduced with permission from Elsevier.

These obstacles notwithstanding, pooled analysis of the outcomes of the levosimendan regulatory trials provided strong indications, albeit not always statistically conclusive proof, of an overall survival benefit (Fig. 5). Extensive experience with levosimendan has been accrued in smaller, often single-center, nonregulatory studies. Many of those studies indicate a survival benefit from levosimendan, a finding affirmed in meta-analysis.^67^

**FIGURE 6.**
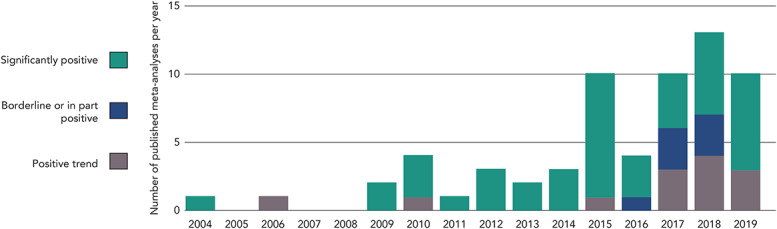
Results of 64 meta-analyses of levosimendan clinical trials. Refer to the supplementary material for details of the individual meta-analyses.

Levosimendan has been evaluated in more than 200 clinical trials during its lifetime, in an extensive range of therapeutic settings. Experience in all those areas has been evaluated in meta-analyses, 31 of which have been conducted in the past 3 years (Fig. 6). In every instance, levosimendan was associated with a favorable impact on the outcomes under consideration but, depending on the data selected, statistical significance in some cases remained elusive. Key therapeutic areas analyzed in this way have included AHF, advanced HF (AdHF), cardiac surgery, and sepsis, all of which have provided indications of benefit from levosimendan therapy.

The broadly affirmative findings of these exercises may be compared with similar appraisals of dobutamine and PDE inhibitors, which have been associated with overall worse mid-to-long-term prognosis.^78–80^ These contrasting findings highlight the distinction between inotropes that act, via either adrenergic or PDE-targeted pathways, to increase intracellular cAMP levels in cardiomyocytes and levosimendan, realizing the ambitions of its inventors by promoting cardiac contractility without compromising the longer-term viability of cardiac muscle cells. This distinction is also illuminated by findings from the Acute Heart Failure Global Survey of Standard Treatment (ALARM-HF) registry, data from which were strongly indicative of survival benefit from levosimendan vis-à-vis adrenergic/calcium-mobilizing inotropes, such as dobutamine.^81^

## LEVOSIMENDAN IN CURRENT USE

The nonunivocal findings of long-term survival benefit from short-term treatment with IV levosimendan have not prevented the drug from establishing itself in the therapeutic repertoire: it has been used in almost 2 million patients since 2000, when its first market authorization was granted by the Swedish regulatory authorities on the basis of the data available at that time.

Its favorable, rapid, and sustained impact on hemodynamics, neurohormone levels, and symptoms in acute decompensated HF are undisputed and of clear therapeutic value. Formal acknowledgement of that value emerged in 2005, when it was mandated in the ESC guidelines.^82^ In the subsequent European guidelines (2008, 2012), the endorsements of levosimendan were more cautious, reflecting a general dissatisfaction of the HF medical community with the concept of inotropy.^83,84^ Levosimendan is currently recommended in the acute treatment of HF to reverse the effect of beta-blockade, if beta-blockade is thought to be contributing to hypotension with subsequent hypoperfusion.^85^

Due to the large therapeutic field they encompass, the European guidelines on acute and chronic HF are not as detailed as they could be and, in recommending therapeutic agents, ignore some of the different etiologies and manifestations of AHF. **Supplemental Digital Content 1** (see **Supplementary Information**, http://links.lww.com/JCVP/A472) and recommendations can be found in more than 20 expert consensus papers coauthored by more than 180 clinicians from 30 countries who have discussed when and how to use levosimendan in different therapeutic settings, including AHF and cardiogenic shock,^74,81,86–88^ AdHF,^89–92^ perioperative use,^93–95^ and use in the intensive care unit (ICU),^96^ and who have described its cardiorenal effects,^88,97^ its effects on quality of life,^98,99^ exercise performance,^100^ lung function,^101^ and pharmacoeconomic considerations.^102^

In the context of a 20-year retrospective, it is worth noting at this point that its complex mode of action might have had the potential to disadvantage levosimendan both in fact and in perception. In fact, that plurality of effects has emerged as both an important aspect of the drug's clinical versatility and usefulness and as a stimulant to informed speculation among experts and to medical research.^73,92,94,96,103^

### Levosimendan in Acute Settings

The most recent ESC guidelines, issued in 2016, identify short-term treatment with IV levosimendan (along with adrenergic inotropes or PDE inhibitors) as an option in the acute-phase management of “patients with hypotension (SBP <90 mmHg) and/or symptoms of hypoperfusion despite adequate filling status, to increase CO, increase blood pressure, improve peripheral perfusion and maintain end-organ function.”^85^ The ESC statement further endorses the short-term use of levosimendan to circumvent the effects of beta-blockade “if beta-blockade is thought to be contributing to hypotension with subsequent hypoperfusion.” The high proportion of patients now receiving beta-blockers as part of the treatment repertoire for chronic HF means that levosimendan has become an important resource in the management of acute decompensations in those patients.

The vasodilator dimension of levosimendan's pharmacology is pertinent to the drug's use in low-output states such as AHF, in which a key pathology is organ hypoperfusion. A drug that both augments CO and improves vasodilatation may be expected to have a more favorable impact in some cases than an agent that acts on CO alone.^104^

In this context, it is important to register that, in many acute settings, hypoperfusion and hypotension may not necessarily be only an effect of inadequate myocardial contractility but may also be related to shock-specific changes in vascular tone and vasodilatation. Thus, besides the need to exclude or correct inadequate volume status, concomitant treatment with a vasopressor agent before embarking on a course of inotropic therapy must also be considered. The importance of correcting inadequate volume before embarking on a course of vasodilatory or inotropic therapy must also be considered and monitored during therapy. These observations may be usefully contextualized into a ‘right patient, right drug’ schema that can be used to guide vasoactive and/or inotropic therapy (Table 3).^105^ The first phase of this schema examines whether or not an inotrope is needed at all and includes exclusion of otherwise treatable causes or the availability of viable alternatives. The next step is to identify the most suitable inotrope: levosimendan figures prominently in several categories, including cardiogenic shock, cardiac surgery, and right ventricular failure.

**TABLE 3.**
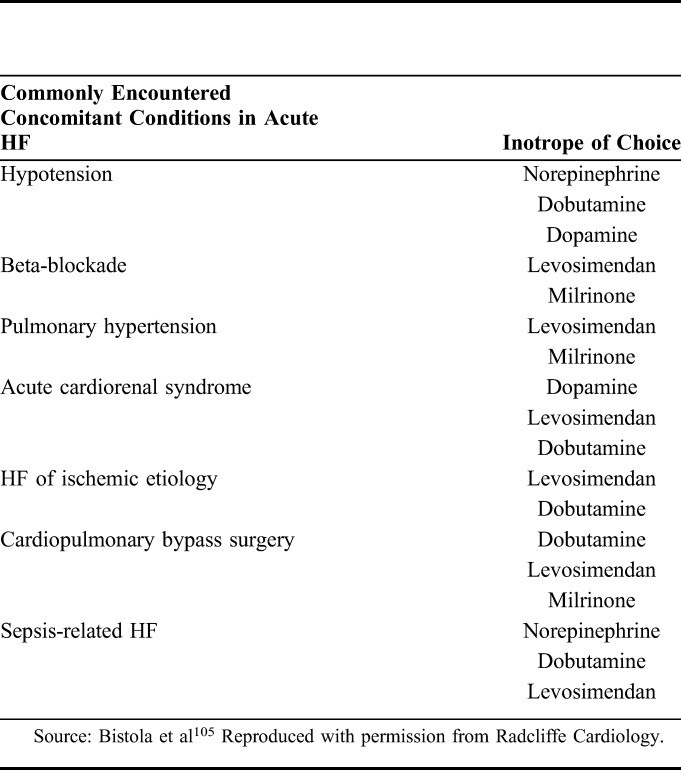
Common Concomitant Conditions in Acute HF and the Corresponding Inotrope of Choice

#### Cardiogenic Shock

The purpose of inotropic support in cardiogenic shock secondary to left ventricular dysfunction is to aid the failing left ventricle by unloading it, increasing left ventricular output, and improving coronary blood flow and hence myocardial perfusion, at the same time decreasing pulmonary edema. The inodilator profile of levosimendan provides a possibly more complete response to those needs than pure inotropic agents, and its ability to promote inotropy with little or no adverse effect on metabolic rate, energy demand, or oxygen consumption may be a bonus. Use of levosimendan may be beneficial in part via a substitution effect in which it reduces the need for catecholaminergic agents, which have less favorable effects on oxygen and energy consumption at the cellular level and a propensity to increase mortality.^106^

Formal experience with levosimendan in cardiogenic shock is limited, but it appears to be generally well tolerated, to improve multiple indices of cardiac function, and to reduce systemic vascular resistance.^106–111^ Levosimendan has also been reported to restore ventriculoarterial coupling and improve left ventricular function in various settings: this may be a further benefit in cardiogenic shock, but this conjecture is currently untested.^112,113^

#### Takotsubo Syndrome

An example of cardiogenic shock in which treatment with levosimendan presents unique benefits is takotsubo syndrome, or stress cardiomyopathy.^114^ Takotsubo-induced HF and/or cardiogenic shock is commonly treated with aggressive diuresis, hemodynamic support, and inotropic drugs. The fact that catecholamines may be implicated in its pathogenesis suggests that catecholamine inotropes may be contraindicated, because these drugs increase cAMP within the cell, increase myocardial oxygen consumption, and may worsen myocardial stunning. Levosimendan, by contrast, as a noncatecholamine inotrope that does not increase myocyte cAMP or oxygen consumption, is a rational therapeutic option in Takotsubo-related cardiogenic shock.^115–117^

#### Cardiac Surgery

Levosimendan has been studied in more than 40 clinical trials in cardiac surgery, with indications emerging that it can reduce the risk of low cardiac output syndrome (LCOS) or be effective in treating postoperative LCOS. The scale of this benefit (derived from a meta-analysis of 14 studies) is moderate but tangible, and is more marked in patients with baseline low left ventricular ejection fraction.^118^

A recalculation incorporating data from 3 recent large RCTs [Levosimendan in Coronary Artery Revascularisation (LICORN), Levosimendan to Reduce Mortality in High Risk Cardiac Surgery Patients (CHEETAH), and LEVO-CTS] causes dilution of the effect size of that estimate.^119–122^ Expert advice derived from these new data is that “levosimendan cannot be at the moment recommended for routine use in all cardiac surgery settings.”^122^ However, there appears to be potential for significant mortality benefit in some subgroups of patients, such as those with low ejection fraction or those undergoing isolated coronary artery bypass grafting (CABG) procedures.^123,124^ Whether these findings may be interpreted as a lack of efficacy of levosimendan in patients undergoing valve surgery due to differences in the underlying pathophysiology or dosing, or as a result of procedure-specific differences in surgical and perfusion management (ie, too low a dose applied before cardiopulmonary bypass or use of crystalloid cardioplegia solutions) needs to be addressed in future studies.

The emphasis on postoperative mortality in these recent studies was substantially driven by regulatory requirements in the design of LEVO-CTS. Whether that outcome is the most relevant or revealing for evaluation of an intervention is an open question. Levosimendan exhibited efficacy in other measures, including a lower incidence of LCOS, less need for rescue catecholamines for inotropic support, and augmentation of the cardiac index.^118^ Methodological difficulties also affected the interpretation of the LICORN and CHEETAH trials.

These caveats notwithstanding, mortality was numerically lower in levosimendan-treated patients in LEVO-CTS.^118^ In addition, the safety profile of levosimendan revealed in all these recent trials identifies it as arguably the safest agent among the broad grouping of inotropes and inodilators. There was no significant excess of arrhythmias or hypotension and no increase in mortality in levosimendan-treated patients.^118^

Encouragement for further evaluation of levosimendan in this area comes from other recent investigations. Wang et al^123^ analyzed data from 21 randomized trials (n = 1727) and calculated that IV levosimendan in patients undergoing CABG was associated with significant reductions in mortality rate (*P* = 0.001) and postoperative atrial fibrillation (*P* = 0.04), with benefit mostly restricted to patients pretreated in advance of an isolated CABG procedure; on-pump status also affected outcomes.^123^ Levosimendan was associated with a higher incidence of hypotension (OR 2.26). These data are consistent with the findings from LEVO-CTS and offer indications of future lines of clinical appraisal.^118,124^ See also Weber et al.^125^

#### Right Ventricular Failure

Determinative randomized trials of levosimendan in right ventricular failure (with or without pulmonary hypertension) have yet to be conducted but a recent meta-analysis of 10 studies of levosimendan in acute right-sided HF identified statistically robust benefits over placebo, with increases in tricuspid annular plane systolic excursion and ejection fraction, plus reductions in systolic pulmonary artery pressure (*P* = 0.0001) and pulmonary vascular resistance (*P* = 0.003).^126^ Adverse events were reported not to differ significantly between groups.

### Effects on Renal Function in HF and Critical Illness

HF is a systemic syndrome involving the kidneys, lungs, and liver, with a great impact on prognosis, and effects of cardiovascular drugs on noncardiac organs are of the utmost importance.^127^ Evidence for a renal-protective action of levosimendan has been reported from preclinical experiments.^128–130^ It has been proposed that levosimendan may cause selective vasodilation on the afferent arterioles of the renal glomeruli, thus improving renal filtration.^97^ This suggestion is compatible with findings from the LIDO trial, in which levosimendan treatment was associated with an increase in the estimated glomerular filtration rate (eGFR) but treatment with dobutamine was not, even though both drugs increased cardiac index and urine output.^63^ It is also consistent with recent reports by Fedele et al^131^ and by Lannemyr et al.^132^ The substantial enhancement of the eGFR observed in the second of those studies was not accompanied by impairment of renal oxygenation, given that renal oxygen delivery increased in proportion to the increase in the eGFR. Nonimpairment of the renal oxygen supply–demand relationship despite eGFR enhancement during levosimendan exposure has also been reported by Bragadottir et al.^133^ A recent report shows that the eGFR enhancement effect of levosimendan is not shared with milrinone.^134^

Data on the effects of levosimendan on renal function in various clinical situations, including cardiac surgery and critical illness, have been collated and the results support a renal-protective effect, making levosimendan the inotrope of choice in the case of worsening cardiorenal syndrome.^135–138^ However, in all these situations, specifically designed prospective trials of adequate statistical power will be needed to confirm the effects and their clinical consequences.

### Levosimendan in AdHF

Adoption of repeated intermittent cycles of IV levosimendan for the treatment of AdHF has been a significant milestone in both the lifecycle of the drug and the management of a complex aspect of HF. Patients with AdHF are on a trajectory ultimately either to a definitive intervention through heart transplantation or the implantation of a left ventricular assist device (LVAD), or to a palliative care pathway. Goals of therapy in AdHF include hemodynamic stabilization and preservation of functional capacity, mitigation of symptoms, and preservation of health-related quality of life. Prevention of HF-related hospitalization is another key goal, both as a desirable outcome per se and as a way of averting the markedly worsened mortality that accompanies hospitalization.^139,140^

All of the pharmacological properties of levosimendan outlined earlier—notably its metabolite-mediated persistence of effect—make it well-suited for repeated or intermittent use in the management of AdHF.

Three randomized, placebo-controlled, double-blind clinical trials, Randomised Trial Investigating the Efficacy and Safety of Pulsed Infusions of Levosimendan in Outpatients with Advanced Heart Failure (Levo-Rep; NCT01065194), Levosimendan Intermittent Administration in Outpatients: Effects on Natriuretic Peptides in Advanced Chronic Heart Failure (LION-HEART; NCT01536132), and Long-Term Intermittent Administration of Levosimendan in Patients with Advanced Heart Failure (LAICA; NCT00988806), have examined the application of repeated cycles of levosimendan therapy in this setting.^141–143^ All these studies demonstrated that repeat-cycle levosimendan reduces N-terminal pro-BNP (NT-proBNP) levels, and there were repeated and clear demonstrations of trends toward reductions in HF readmissions and mortality that are consistent with, and corroborate, the findings of meta-analyses.^144,145^ A recognized overall conclusion from these studies is that repetitive application of levosimendan is feasible and safe in an outpatient setting.^141–143^ Notably, onset destabilization is not invariably an immediate-onset event, however, and it may be possible to identify opportunities when timely recognition of—and intervention on—signs and symptoms of decompensation may avoid unplanned/urgent hospitalizations due to hemodynamic crises.

The need for a larger randomized study (or studies) in this area is being addressed by the Repetitive Levosimendan Infusion for Patients with Advanced Chronic Heart Failure trial (LEODOR; NCT03437226), a multicenter, randomized, double-blind, placebo-controlled, 3-arm trial, which will examine the impact and safety of intermittent levosimendan therapy, started during the vulnerable phase after a recent hospitalization for HF.^146^ Treatment effectiveness will be assessed using a hierarchical composite clinical end point consisting of time to death or urgent heart transplantation or implantation of a ventricular assist device; time to nonfatal HF hospitalization requiring IV vasoactive therapy; and time-averaged proportional change in NT-proBNP. Basing the trial on such an outcome measure should enhance the power to examine whether, compared with placebo, repeated use of levosimendan is associated with greater clinical stability over the course of subsequent weeks.

In addition to its use in maintaining hemodynamic stability in patients with AdHF, preoperative use of IV levosimendan in patients undergoing implantation of an LVAD, or identification of LVAD candidates, has been reported to be “generally well-tolerated and not interrupted because of side effects” and associated with significant improvements in end-organ function, although with similar early mortality rates.^147^ This is an application where a substantial expansion in the use of levosimendan may be anticipated.

The current clinical applications of IV levosimendan are summarized in Table 4.

**TABLE 4.**
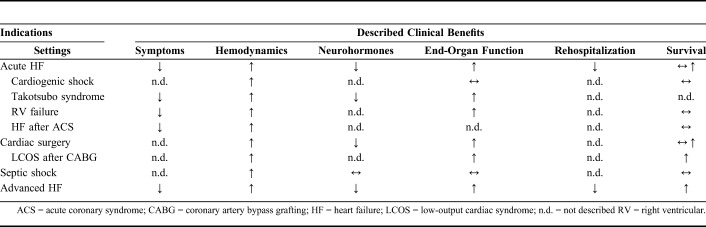
Current Clinical Applications of IV Levosimendan

## THE NEXT 20 YEARS OF LEVOSIMENDAN

In addition to the LEODOR study, there are currently more than 20 investigator-initiated clinical trials in progress in which levosimendan is being evaluated for possible therapeutic benefits. These include: a study designed to examine the effect of a single 24-hour infusion of levosimendan to prevent rehospitalization in patients with severe systolic HF (NCT03764722); Effect of Levosimendan or Placebo on Exercise in Advanced Chronic Heart Failure (LOCO-CHF; NCT03576677), a placebo-controlled appraisal of the effects of IV levosimendan on exercise capacity in patients with advanced chronic HF; and research into the effects of levosimendan on acute kidney injury after cardiac surgery (LEVOAKI; NCT02531724). The Early Management Strategies of Acute Heart Failure for Patients with NSTEMI study (EMSAHF; NCT03189901) is exploring whether early use of levosimendan in patients with acute MI combined with elevated BNP/NT-proBNP may reduce the risk of emergent AHF and improve outcome.

Developments in the technology and science of telemedicine and telemonitoring may make this a practical proposition in the foreseeable future. The ambition (already under active exploration and with progress further accelerated by the introduction of artificial intelligence into the diagnostic loop) is to develop patient monitoring to such a degree of immediacy and accuracy that overt decompensations may be wholly avoided by prompt, appropriate clinical responses to the first signs of deterioration.^148,149^ Intermittent IV levosimendan may be an appropriate intervention in this ‘acute but nonhospitalized’ scenario, depending on the clinical circumstances of an individual patient. Investigations in this direction can be expected, although these are likely to be driven primarily by developments in telemedicine technologies, rather than by any focus on specific medical interventions.^150^

A range of ICU situations has been identified in which levosimendan may offer clinical benefits, either as an adjunct to existing interventions or as an alternative to conventional therapies. These situations include: hemodynamic support in cardiac critical care,^151^ hemodynamic support in septic cardiomyopathy,^152,153^ weaning from the ventilator,^154^ weaning from venoarterial extracorporeal membrane oxygenation after cardiac surgery,^155–159^ and renal failure and kidney protection in cardiorenal syndrome.^97,130,132,160–166^

In several of these areas, notably low CO syndrome, cardiogenic shock, Takotsubo cardiomyopathy, and sepsis, a substantial element of any benefit accruing from use of levosimendan may be attributable to the substitution of a nonadrenergic stimulant for conventional catecholaminergic agents, such as dobutamine, thereby averting some of the potential toxic complications of adrenergic overstimulation.^167^

‘Decatecholaminization’ of the medical armamentarium is a developing line of practice in the management of critically ill patients.^168–176^ As an established nonadrenergic vasoactive agent that offers positive cardiovascular effects (eg, ventriculoarterial recoupling, decongestion), as well as potentially advantageous ancillary effects on kidney function and cellular-protective actions, levosimendan is both a therapeutic resource and experimental tool for investigating this new approach.^161,177–184^

Developments in these areas, although already pioneered by promising exploratory studies, will, inevitably, require well-designed and properly powered RCTs.^151–153,185,186^

Central to future investigations will be the identification of robust and relevant end points. An overemphasis on crude mortality may not be the most informative metric by which to judge outcomes. Trials directed toward establishing the best overall therapeutic strategy may be more progressive than studies framed to position any one agent as the best for a particular purpose or situation. Various commentaries have examined the challenges of conducting clinical trials in critically ill patients.^187–190^ Of notable significance in this respect is the work of Mebazaa et al,^191^ who have sought to apply lessons learned from trials in AHF to the more flexible and less prescriptive design of phase III studies in sepsis.

Principal among those lessons is the need to move toward composite primary end points, with isolated all-cause mortality addressed as a safety signal, not a primary marker of effectiveness. From that perspective, the model of HF as a complex condition of multifactorial causes and pathophysiology, and one therefore unlikely to be moderated by a single intervention on a simple outcome measure such as total mortality, is an example of a needlessly restrictive approach to clinical trials design that has had a dampening effect on the development of needed therapies. In addition, there has been recent discussion about how syndrome-attributable risks of critical illness-associated diagnoses have often likely been overestimated using common statistical methods, contributing to very low success rates in this field.^192^ The authors of this article are unanimously supportive of that view, which we feel has frustrated advances in HF care. It is to be hoped that our colleagues in cardiac critical care are able to benefit in the design of their own clinical trials by examining some of the methodological missteps and misplaced emphases that have hampered some aspects of cardiology research in recent decades.

Levosimendan has been approved by Chinese authorities on the basis of several local corroborative regulatory clinical trials, and SIMDAX will enter that important market this year.^161,193–195^

The use of levosimendan in pediatric patients is currently contraindicated due to a lack of regulatory studies. A few investigator-initiated studies have been performed. The largest published study included retrospectively gathered data on 484 levosimendan infusions delivered to 293 patients at a single pediatric ICU; the majority of the patients (65%) were aged 12 months or younger.^196^ Levosimendan postponed or reduced the need for mechanical cardiac support in children with cardiomyopathy or who were undergoing cardiac surgery. In other reports, levosimendan was compared to the PDE inhibitor milrinone and found to be either similarly efficacious or superior.^197–199^ In a randomized double-blind study in children younger than 4 years of age undergoing cardiac surgery, patients receiving levosimendan had significantly higher cardiac index and lower pulmonary artery pressure than children receiving dobutamine.^200^ There is a strong rationale to perform properly powered clinical regulatory trials on the pediatric use of levosimendan in the near future.

### Research and Development Outside Cardiology

#### Respiratory Function

Respiratory muscle dysfunction may develop in the course of several diseases, including chronic obstructive pulmonary disease, HF, and critical illness.^201,202^ In addition to atrophy, reduced calcium sensitivity of contraction plays an important role in respiratory muscle weakness in these conditions.^203,204^ Levosimendan has been studied in noncardiac muscle, especially in respiratory muscles. In vitro studies have demonstrated that levosimendan improves calcium sensitivity of force generation in diaphragm fibers from healthy subjects and patients with chronic obstructive pulmonary disease.^203^ In a physiological study, levosimendan at a clinically used dose improved diaphragm contractile efficiency by 21% and reversed diaphragm fatigue induced by inspiratory muscle loading.^205^ In a placebo-controlled randomized study, the effects of levosimendan were evaluated in critically ill patients being weaned from mechanical ventilation.^206^ There was no difference in the primary end point of contractile efficiency between groups, although tidal volume and minute ventilation were both significantly higher in the levosimendan group, and arterial CO_2_ tension significantly lower.^206^ Improved respiratory mechanics may facilitate liberation from mechanical ventilation, although this requires further clinical studies.

#### Pulmonary Hypertension

A phase II regulatory clinical trial is currently underway in the United States on the repeated use of IV levosimendan for pulmonary hypertension in patients with HF and preserved ejection fraction (NCT03541603). The results are expected in 2020.

#### Motor Neuron Disease

There is interest in the potential of oral levosimendan in the management of amyotrophic lateral sclerosis (ALS). ALS is characterized by a progressive muscular paralysis arising from motor neuron degeneration.^207^ The disease eventually involves most skeletal muscles, plus the diaphragm and other respiratory muscles, leading to death from respiratory failure.^208,209^ It is the most common neurodegenerative disorder of mid-life, with incidence and prevalence increasing with age.^210^ ALS is currently incurable, and the medical options are limited. No treatment is currently approved to enhance motor function in ALS, and recent clinical experiences have produced mixed results.^211–214^

The physiological and pharmacological rationale for levosimendan in ALS rests on the fact that both the diaphragm and skeletal muscle express genes for the slow-twitch (or cardiac) isoform of TnC (the diaphragm consists of approximately 50% slow-twitch fibers).^215^ As a calcium sensitizer with cTnC as molecular target, levosimendan can thus strengthen contractility also in the diaphragm and skeletal muscle. The multifaceted pharmacology of levosimendan may also provide supplementary clinical impact in patients with ALS through the already-mentioned range of pharmacological effects not directly related to the drug's calcium-sensitizing action.^37,216^

Positive effects of short-term oral levosimendan in patients with ALS were seen in the Effects of ODM-109 on Respiratory Function in Patients With ALS (LEVALS; NCT02487407) study, a phase II trial that used a randomized, double-blind, placebo-controlled, cross-over design to evaluate the efficacy and safety of oral levosimendan in patients with definite or probable ALS. The 66 patients enrolled had experienced symptoms of ALS for between 12 and 48 months and had an early decline in respiratory function. Therapy consisted of 2 weeks of oral levosimendan at doses of 1 or 2 mg/d or placebo, administered in random order during 3 study periods separated by a wash-out period.^217^

The phase III Effects of Oral Levosimendan (ODM-109) on Respiratory Function in Patients with ALS trial (REFALS; NCT03505021) is ongoing in North America, Europe, and Australia. An open-extension phase of REFALS should offer important insights into the long-term safety and efficacy of oral levosimendan in ALS.

#### Translational and Early Development Phases

Other trials are looking at the effects of IV levosimendan on cellular metabolic alterations in patients with septic shock (NCT02963454) and the possibility that IV levosimendan may improve the prognosis in acute respiratory distress syndrome (NCT04020003).

Other recent research on the effect of levosimendan on oxidative stress in a mouse model of diabetes showing an effect of the drug in preventing memory impairment has opened up a new possible development path.^218,219^ A separate report, again in mice, highlighted the protective effects elicited by levosimendan against liver ischemia/reperfusion injury.^220^

Of particular interest, new pharmaceutical agents are currently being developed with levosimendan and cTnC as a pharmacophore model in unexpected fields such as oncology.^221^

## CONCLUSIONS

In the field of short-term hemodynamic treatments for acute cardiac care, levosimendan represents a rare case of an inotrope approved by regulatory authorities in the past 20 years. The approval was based on data from 3 phase III clinical studies in which the end points were reached (LIDO, RUSSLAN, and REVIVE).^62,63,65^ The safety data collected during those 20 years of clinical use are unprecedented and superior to those for any of the other inotropes or inodilators.^222^ In a recent consensus paper, the authors evaluated whether “the nearly total absence of evidence of benefit with some of the traditional IV drugs used in AHF and AdHF (such as the catecholamines or the PDE inhibitors) would warrant their elimination from routine use in favor of treatments where such evidence has been accrued (e.g. for levosimendan).”^75^ With regard to posology, both a frequent use in the therapy of AdHF and an earlier use of levosimendan in the therapy of AHF have been shown to be of benefit.^91,223^ Beyond that, 20 years after its initial approval for clinical use, levosimendan remains an important resource in cardiovascular medicine and a valuable tool for clinical research, investigation, and innovation in that and other areas of medicine.^20^ The clinical program for the development of oral levosimendan as a treatment for ALS shows how retargeting a safe drug, even in a different formulation, is a rational strategy in pharmaceutical development.^224^

## Supplementary Material

SUPPLEMENTARY MATERIAL
