# Supplementary material for: Levosimendan Efficacy and Safety: 20 Years of SIMDAX in Clinical Use
Source: J Cardiovasc Pharmacol. 2020 Jul 6;76(1):4–22. doi: 10.1097/FJC.0000000000000859 (PMC7340234; doi:10.1097/FJC.0000000000000859)
Supplement: SUPPLEMENTARY MATERIAL [file jcvp-76-04-s001.docx]

**Supplementary materials**

**Supplementary Table 1:** results of 64 meta-analyses on levosimendan clinical trials in specified clinical scenarios from 2004 to 2019. RR, risk reduction; RD, relative difference; OR, odds ratio; SMD, standard mean difference; WMD, weighted mean difference; CI, cardiac index; ^@^network meta-analysis; hemodyn., hemodynamic effects; SOC, Standard of Care; topSOC, on top of Standard of Care; dobutam., dobutamine; CHF, congestive heart failure; CABG, coronary artery bypass surgery; LCOS, low cardiac output syndrome; LVEF, left ventricular ejection fraction; AKI, acute kidney injury; AdHF, Advanced Heart Failure; re-hosp, re-hospitalization for acute heart failure; ACS-HF, heart failure complicating acute coronary syndrome; SIT, standard inotropic therapy; ARF, acute renal failure; RRT, renal replacement therapy; BNP. brain natriuretic peptide; cTn peak, postoperative release of cardiac troponin. ^a^trial sequential analysis (TSA), non-unequivocal; ^b^Bayesian analysis; ^c^patients with cardiogenic shock and/or low cardiac output syndrome; ^d^patients with cardiogenic shock complicating myocardial infarction; ^e^in patients with severely reduced left ventricular systolic function; ^f^in a sub-analysis of low bias RCTs only, the significance was lost; ^g^long term mortality for patients with LVEF≤30%, as from the paper supplements (when all the 5 studies with long term mortality data were considered, the significance was lost); ^h^paediatric settings; ^i^meta-analysis of the regulatory studies described in the authorization dossier, the cumulative data on mortality show a borderline statistical significance (p=0.054); ^j^pooled analysis to study the influence of concurrent sulfonylurea treatment to the levosimendan effects; ^k^in the same meta-analysis, a comparison of levosimendan versus placebo on 6 studies and 1678 patients yielded an OR of 0.83 in favour of levosimendan without reaching formal statistical significance.

**
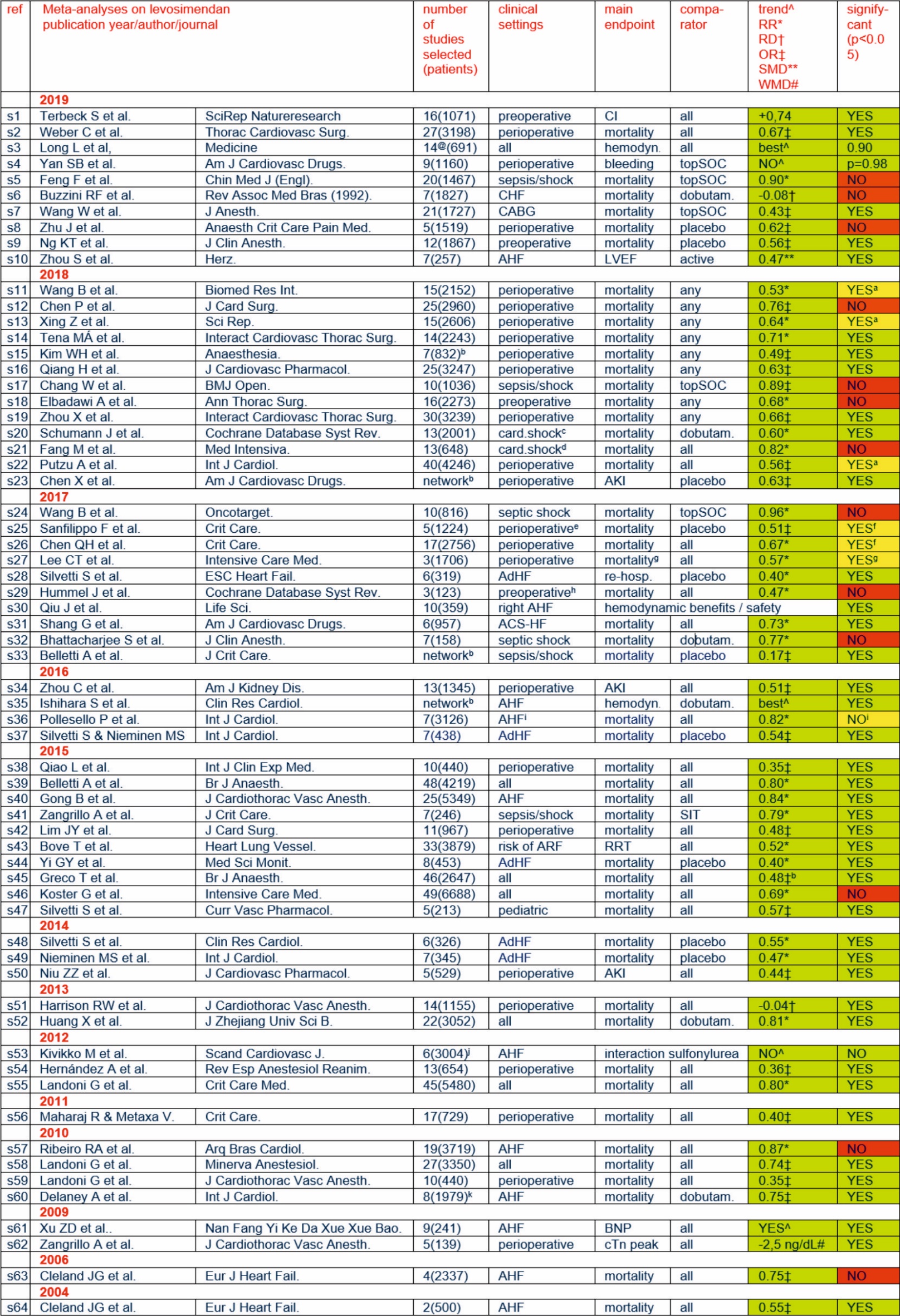
**

**References to supplementary materials:**

s1. Terbeck S, Heinisch PP, Lenz A, Friess J-O, Guensch D, Carrel T, Eberle B, Erdoes G. Levosimendan and systemic vascular resistance in cardiac surgery patients: a systematic review and meta-analysis. SciRep Natureresearch 2019;9:20343. doi:10.1038/s41598-019-56831-y

s2. Weber C, Esser M, Eghbalzadeh K, Sabashnikov A, Djordjevic I, Maier J, Merkle J, Choi YH, Madershahian N, Liakopoulos O, Deppe AC, Wahlers TCW. Levosimendan Reduces Mortality and Low Cardiac Output Syndrome in Cardiac Surgery. Thorac Cardiovasc Surg. 2019 Nov 26. doi: 10.1055/s-0039-3400496; PMID: 31770777

s3. Long L, Zhao H, Shen L, He C, Ren S, Zhao H. Hemodynamic effects of inotropic drugs in heart failure: a network meta-analysis of clinical trials. Medicine. 2019;98(47):e18144. doi: 10.1097/MD.0000000000018144.

s4. Yan SB, Wang XY, Shang GK, Wang ZH, Deng QM, Song JW, Sai WW, Song M, Zhong M, Zhang W. Impact of Perioperative Levosimendan Administration on Risk of Bleeding After Cardiac Surgery: A Meta-analysis of Randomized Controlled Trials. Am J Cardiovasc Drugs. 2019 Sep 16. doi: 10.1007/s40256-019-00372-2; PMID: 31523760.

s5. Feng F, Chen Y, Li M, Yuan JJ, Chang XN, Dong CM. Levosimendan does not reduce the mortality of critically ill adult patients with sepsis and septic shock: a meta-analysis. Chin Med J (Engl). 2019 May 20;132(10):1212-1217. doi: 10.1097/CM9.0000000000000197; PMID: 31140992

s6. Buzzini RF, Silvinato A, Floriano I, Bernardo WM, Almeida GR. Decompensated congestive heart failure - treatment with levosimendan. Rev Assoc Med Bras (1992). 2019 May 2;65(4):524-529. doi: 10.1590/1806-9282.65.4.524; PMID: 31066804.

s7. Wang W, Zhou X, Liao X, Liu B, Yu H. The efficacy and safety of prophylactic use of levosimendan on patients undergoing coronary artery bypass graft: a systematic review and meta-analysis. J Anesth. 2019 Aug;33(4):543-550. doi: 10.1007/s00540-019-02643-3; PMID: 31025104.

s8. Zhu J, Zhang Y, Chen L, He Y, Qing X. Levosimendan in patients with low cardiac output syndrome undergoing cardiac surgery: A systematic review and meta-analysis. Anaesth Crit Care Pain Med. 2019 Jun;38(3):243-249. doi: 10.1016/j.accpm.2018.08.005; PMID: 30342103.

s9. Ng KT, Chan XL, Tan W, Wang CY. Levosimendan use in patients with preoperative low ejection fraction undergoing cardiac surgery: A systematic review with meta-analysis and trial sequential analysis. J Clin Anesth. 2019 Feb;52:37-47. doi: 10.1016/j.jclinane.2018.08.019; PMID: 30172838.

s10. Zhou S, Zhang L, Li J. Effect of levosimendan in patients with acute decompensated heart failure : A meta-analysis. Herz. 2019 Nov;44(7):630-636. doi: 10.1007/s00059-018-4693-3; PMID: 29637229.

s11. Wang B, He X, Gong Y, Cheng B. Levosimendan in Patients with Left Ventricular Dysfunction Undergoing Cardiac Surgery: An Update Meta-Analysis and Trial Sequential Analysis. Biomed Res Int. 2018 May 8;2018:7563083. doi: 10.1155/2018/7563083. eCollection 2018; PMID: 29854789

s12. Chen P, Wu X, Wang Z, Li Z, Tian X, Wang J, Yan T. Effects of levosimendan on mortality in patients undergoing cardiac surgery: A systematic review and meta-analysis. J Card Surg. 2018 Jun;33(6):322-329. doi: 10.1111/jocs.13716; PMID: 29785788.

s13. Xing Z, Tang L, Chen P, Huang J, Peng X, Hu X. Levosimendan in patients with left ventricular dysfunction undergoing cardiac surgery: a meta-analysis and trial sequential analysis of randomized trials. Sci Rep. 2018 May 17;8(1):7775. doi: 10.1038/s41598-018-26206-w. PMID: 29773835

s14. Tena MÁ, Urso S, González JM, Santana L, Sadaba R, Juarez P, González L, Portela F. Levosimendan versus placebo in cardiac surgery: a systematic review and meta-analysis. Interact Cardiovasc Thorac Surg. 2018 Nov 1;27(5):677-685. doi: 10.1093/icvts/ivy133; PMID: 29718383.

s15. Kim WH, Hur M, Park SK, Jung DE, Kang P, Yoo S, Bahk JH. Pharmacological interventions for protecting renal function after cardiac surgery: a Bayesian network meta-analysis of comparative effectiveness. Anaesthesia. 2018 Aug;73(8):1019-1031. doi: 10.1111/anae.14227; PMID: 29682727.

s16. Qiang H, Luo X, Huo JH, Wang ZQ. Perioperative Use of Levosimendan Improves Clinical Outcomes in Patients After Cardiac Surgery: A Systematic Review and Meta-Analysis. J Cardiovasc Pharmacol. 2018 Jul;72(1):11-18. doi: 10.1097/FJC.0000000000000584; PMID: 29672418.

s17. Chang W, Xie JF, Xu JY, Yang Y. Effect of levosimendan on mortality in severe sepsis and septic shock: a meta-analysis of randomised trials. BMJ Open. 2018 Mar 30;8(3):e019338. doi: 10.1136/bmjopen-2017-019338; PMID: 29602841

s18. Elbadawi A, Elgendy IY, Saad M, Megaly M, Mentias A, Abuzaid AS, Shahin HI, Goswamy V, Abowali H, London B. Meta-Analysis of Trials on Prophylactic Use of Levosimendan in Patients Undergoing Cardiac Surgery. Ann Thorac Surg. 2018 May;105(5):1403-1410. doi: 10.1016/j.athoracsur.2017.11.027; PMID: 29573810.

s19. Zhou X, Hu C, Xu Z, Liu P, Zhang Y, Sun L, Wang Y, Gao X. Effect of levosimendan on clinical outcomes in adult patients undergoing cardiac surgery: a meta-analysis of randomized controlled trials. Interact Cardiovasc Thorac Surg. 2018 Jun 1;26(6):1016-1026. doi: 10.1093/icvts/ivy017; PMID: 29415177.

s20. Schumann J, Henrich EC, Strobl H, Prondzinsky R, Weiche S, Thiele H, Werdan K, Frantz S, Unverzagt S. Inotropic agents and vasodilator strategies for the treatment of cardiogenic shock or low cardiac output syndrome. Cochrane Database Syst Rev. 2018 Jan 29;1:CD009669. doi: 10.1002/14651858.CD009669.pub3; PMID: 29376560.

s21. Fang M, Cao H, Wang Z. Levosimendan in patients with cardiogenic shock complicating myocardial infarction: A meta-analysis. Med Intensiva. 2018 Oct;42(7):409-415. doi: 10.1016/j.medin.2017.08.009; PMID: 29126662.

s22. Putzu A, Clivio S, Belletti A, Cassina T. Perioperative levosimendan in cardiac surgery: A systematic review with meta-analysis and trial sequential analysis. Int J Cardiol. 2018 Jan 15;251:22-31. doi: 10.1016/j.ijcard.2017.10.077; PMID: 29126653.

s23. Chen X, Huang T, Cao X, Xu G. Comparative Efficacy of Drugs for Preventing Acute Kidney Injury after Cardiac Surgery: A Network Meta-Analysis. Am J Cardiovasc Drugs. 2018 Feb;18(1):49-58. doi: 10.1007/s40256-017-0245-0; PMID: 28819767.

s24. Wang B, Chen R, Guo X, Zhang W, Hu J, Gong Y, Cheng B. Effects of levosimendan on mortality in patients with septic shock: systematic review with meta-analysis and trial sequential analysis. Oncotarget. 2017 Aug 10;8(59):100524-100532. doi: 10.18632/oncotarget.20123, PMID: 29245998;

s25. Sanfilippo F, Knight JB, Scolletta S, Santonocito C, Pastore F, Lorini FL, Tritapepe L, Morelli A, Arcadipane A. Levosimendan for patients with severely reduced left ventricular systolic function and/or low cardiac output syndrome undergoing cardiac surgery: a systematic review and meta-analysis. Crit Care. 2017 Oct 19;21(1):252. doi: 10.1186/s13054-017-1849-0; PMID: 29047417;

s26. Chen QH, Zheng RQ, Lin H, Shao J, Yu JQ, Wang HL. Effect of levosimendan on prognosis in adult patients undergoing cardiac surgery: a meta-analysis of randomized controlled trials. Crit Care. 2017 Oct 17;21(1):253. doi: 10.1186/s13054-017-1848-1; PMID: 29041948.

s27. Lee CT, Lin YC, Yeh YC, Chen TL, Chen CY. Effects of levosimendan for perioperative cardiovascular dysfunction in patients receiving cardiac surgery: a meta-analysis with trial sequential analysis. Intensive Care Med. 2017 Dec;43(12):1929-1930. doi: 10.1007/s00134-017-4927-5; PMID: 28884347.

s28. Silvetti S, Belletti A, Fontana A, Pollesello P. Rehospitalization after intermittent levosimendan treatment in advanced heart failure patients: a meta-analysis of randomized trials. ESC Heart Fail. 2017 Nov;4(4):595-604. doi: 10.1002/ehf2.12177; PMID: 28834396;

s29. Hummel J, Rücker G, Stiller B. Prophylactic levosimendan for the prevention of low cardiac output syndrome and mortality in paediatric patients undergoing surgery for congenital heart disease. Cochrane Database Syst Rev. 2017 Aug 2;8:CD011312. doi: 10.1002/14651858.CD011312.pub3; PMID: 28770972;

s30. Qiu J, Jia L, Hao Y, Huang S, Ma Y, Li X, Wang M, Mao Y. Efficacy and safety of levosimendan in patients with acute right heart failure: A meta-analysis. Life Sci. 2017 Sep 1;184:30-36. doi: 10.1016/j.lfs.2017.07.001; PMID: 28689804.

s31. Shang G, Yang X, Song D, Ti Y, Shang Y, Wang Z, Tang M, Zhang Y, Zhang W, Zhong M. Effects of Levosimendan on Patients with Heart Failure Complicating Acute Coronary Syndrome: A Meta-Analysis of Randomized Controlled Trials. Am J Cardiovasc Drugs. 2017 Dec;17(6):453-463. doi: 10.1007/s40256-017-0237-0; PMID: 28597399.

s32. Bhattacharjee S, Soni KD, Maitra S, Baidya DK. Levosimendan does not provide mortality benefit over dobutamine in adult patients with septic shock: A meta-analysis of randomized controlled trials. J Clin Anesth. 2017 Jun;39:67-72. doi: 10.1016/j.jclinane.2017.03.011; PMID: 28494911.

s33. Belletti A, Benedetto U, Biondi-Zoccai G, Leggieri C, Silvani P, Angelini GD, Zangrillo A, Landoni G. The effect of vasoactive drugs on mortality in patients with severe sepsis and septic shock. A network meta-analysis of randomized trials. J Crit Care. 2017 Feb;37:91-98. doi: 10.1016/j.jcrc.2016.08.010; PMID: 27660923.

s34. Zhou C, Gong J, Chen D, Wang W, Liu M, Liu B. Levosimendan for Prevention of Acute Kidney Injury After Cardiac Surgery: A Meta-analysis of Randomized Controlled Trials. Am J Kidney Dis. 2016 Mar;67(3):408-16. doi: 10.1053/j.ajkd.2015.09.015; PMID: 26518388.

s35. Ishihara S, Gayat E, Sato N, Arrigo M, Laribi S, Legrand M, Placido R, Manivet P, Cohen-Solal A, Abraham WT, Jessup M, Mebazaa A. Similar hemodynamic decongestion with vasodilators and inotropes: systematic review, meta-analysis, and meta-regression of 35 studies on acute heart failure. Clin Res Cardiol. 2016 Dec;105(12):971-980. PMID: 27314418.

s36. Pollesello P, Parissis J, Kivikko M, Harjola VP. Levosimendan meta-analyses: Is there a pattern in the effect on mortality? Int J Cardiol. 2016 Apr 15;209:77-83. doi: 10.1016/j.ijcard.2016.02.014; PMID: 26882190.

s37. Silvetti S, Nieminen MS. Repeated or intermittent levosimendan treatment in advanced heart failure: An updated meta-analysis. Int J Cardiol. 2016 Jan 1;202:138-43. doi: 10.1016/j.ijcard.2015.08.188; PMID: 26386941.

s38. Qiao L, Xu C, Li X, Li F, Liu W. Heart calcium sensitizer on morbidity and mortality of high-risk surgical patients with MODS: systematic review and meta-analysis. Int J Clin Exp Med. 2015 Oct 15;8(10):17712-20; PMID: 26770361.

s39. Belletti A, Castro ML, Silvetti S, Greco T, Biondi-Zoccai G, Pasin L, Zangrillo A, Landoni G. The Effect of inotropes and vasopressors on mortality: a meta-analysis of randomized clinical trials. Br J Anaesth. 2015 Nov;115(5):656-75. doi: 10.1093/bja/aev284; PMID: 26475799.

s40. Gong B, Li Z, Yat Wong PC. Levosimendan Treatment for Heart Failure: A Systematic Review and Meta-Analysis. J Cardiothorac Vasc Anesth. 2015 Dec;29(6):1415-25. doi: 10.1053/j.jvca.2015.03.023; PMID: 26275522.

s41. Zangrillo A, Putzu A, Monaco F, Oriani A, Frau G, De Luca M, Di Tomasso N, Bignami E, Lomivorotov V, Likhvantsev V, Landoni G. Levosimendan reduces mortality in patients with severe sepsis and septic shock: A meta-analysis of randomized trials. J Crit Care. 2015 Oct;30(5):908-13. doi: 10.1016/j.jcrc.2015.05.017; PMID: 26093802.

s42. Lim JY, Deo SV, Rababa'h A, Altarabsheh SE, Cho YH, Hang D, McGraw M, Avery EG, Markowitz AH, Park SJ. Levosimendan Reduces Mortality in Adults with Left Ventricular Dysfunction Undergoing Cardiac Surgery: A Systematic Review and Meta-analysis. J Card Surg. 2015 Jul;30(7):547-54. doi: 10.1111/jocs.12562; PMID: 25989324.

s43. Bove T, Matteazzi A, Belletti A, Paternoster G, Saleh O, Taddeo D, Dossi R, Greco T, Bradic N, Husedzinovic I, Nigro Neto C, Lomivorotov VV, Calabrò MG. Beneficial impact of levosimendan in critically ill patients with or at risk for acute renal failure: a meta-analysis of randomized clinical trials. Heart Lung Vessel. 2015;7(1):35-46. PMID: 25861589.

s44. Yi GY, Li JX, Zhang J, Niu LL, Zhang CY. Repetitive infusion of levosimendan in patients with chronic heart failure: a meta-analysis. Med Sci Monit. 2015 Mar 26;21:895-901. doi: 10.12659/MSM.893736; PMID: 25811545.

s45. Greco T, Calabrò MG, Covello RD, Greco M, Pasin L, Morelli A, Landoni G, Zangrillo A. A Bayesian network meta-analysis on the effect of inodilatory agents on mortality. Br J Anaesth. 2015 May;114(5):746-56. doi: 10.1093/bja/aeu446; PMID: 25652947.

s46. Koster G, Wetterslev J, Gluud C, Zijlstra JG, Scheeren TW, van der Horst IC, Keus F. Effects of levosimendan for low cardiac output syndrome in critically ill patients: systematic review with meta-analysis and trial sequential analysis. Intensive Care Med. 2015 Feb;41(2):203-21. doi: 10.1007/s00134-014-3604-1; PMID: 25518953.

s47. Silvetti S, Silvani P, Azzolini ML, Dossi R, Landoni G, Zangrillo A. A systematic review on levosimendan in paediatric patients. Curr Vasc Pharmacol. 2015;13(1):128-33. PMID: 25440597.

s48. Silvetti S, Greco T, Di Prima AL, Mucchetti M, de Lurdes CM, Pasin L, Scandroglio M, Landoni G, Zangrillo A. Intermittent levosimendan improves mid-term survival in chronic heart failure patients: meta-analysis of randomised trials. Clin Res Cardiol. 2014 Jul;103(7):505-13. doi: 10.1007/s00392-013-0649-z; PMID: 24368740.

s49. Nieminen MS, Altenberger J, Ben-Gal T, Böhmer A, Comin-Colet J, Dickstein K, Edes I, Fedele F, Fonseca C, García-González MJ, Giannakoulas G, Iakobishvili Z, Jääskeläinen P, Karavidas A, Kettner J, Kivikko M, Lund LH, Matskeplishvili ST, Metra M, Morandi F, Oliva F, Parkhomenko A, Parissis J, Pollesello P, Pölzl G, Schwinger RH, Segovia J, Seidel M, Vrtovec B, Wikström G. Repetitive use of levosimendan for treatment of chronic advanced heart failure: clinical evidence, practical considerations, and perspectives: an expert panel consensus. Int J Cardiol. 2014 Jun 15;174(2):360-7. doi: 10.1016/j.ijcard.2014.04.111; PMID: 24780540.

s50. Niu ZZ, Wu SM, Sun WY, Hou WM, Chi YF. Perioperative levosimendan therapy is associated with a lower incidence of acute kidney injury after cardiac surgery: a meta-analysis. J Cardiovasc Pharmacol. 2014 Feb;63(2):107-12. doi: 10.1097/FJC.0000000000000028. PMID: 24126568.

s51. Harrison RW, Hasselblad V, Mehta RH, Levin R, Harrington RA, Alexander JH. Effect of levosimendan on survival and adverse events after cardiac surgery: a meta-analysis. J Cardiothorac Vasc Anesth. 2013 Dec;27(6):1224-32. doi: 10.1053/j.jvca.2013.03.027; PMID: 24050857.

s52. Huang X, Lei S, Zhu MF, Jiang RL, Huang LQ, Xia GL, Zhi YH. Levosimendan versus dobutamine in critically ill patients: a meta-analysis of randomized controlled trials. J Zhejiang Univ Sci B. 2013 May;14(5):400-15. doi: 10.1631/jzus.B1200290; PMID: 23645177.

s53. Kivikko M, Nieminen MS, Pollesello P, Pohjanjousi P, Colucci WS, Teerlink JR, Mebazaa A. The clinical effects of levosimendan are not attenuated by sulfonylureas. Scand Cardiovasc J. 2012 Dec;46(6):330-8. doi: 10.3109/14017431.2012.725206; PMID: 22928945.

54. Hernández A, Miranda A, Parada A. [Levosimendan reduces mortality in cardiac surgery: a systematic review and meta-analysis]. Rev Esp Anestesiol Reanim. 2012 Jan;59(1):6-11. doi: 10.1016/j.redar.2012.02.001; PMID: 22429630.

s55. Landoni G, Biondi-Zoccai G, Greco M, Greco T, Bignami E, Morelli A, Guarracino F, Zangrillo A. Effects of levosimendan on mortality and hospitalization. A meta-analysis of randomized controlled studies. Crit Care Med. 2012 Feb;40(2):634-46. doi: 10.1097/CCM.0b013e318232962a; PMID: 21963578.

s56. Maharaj R, Metaxa V. Levosimendan and mortality after coronary revascularisation: a meta-analysis of randomised controlled trials. Crit Care. 2011 Jun 8;15(3):R140. doi: 10.1186/cc10263; PMID: 21651806.

s57. Ribeiro RA, Rohde LE, Polanczyk CA. Levosimendan in acute decompensated heart failure: systematic review and meta-analysis. Arq Bras Cardiol. 2010 Aug;95(2):230-7. PMID: 20549133.

s58. Landoni G, Mizzi A, Biondi-Zoccai G, Bignami E, Prati P, Ajello V, Marino G, Guarracino F, Zangrillo A. Levosimendan reduces mortality in critically ill patients. A meta-analysis of randomized controlled studies. Minerva Anestesiol. 2010 Apr;76(4):276-86. PMID: 20332741.

s59. Landoni G, Mizzi A, Biondi-Zoccai G, Bruno G, Bignami E, Corno L, Zambon M, Gerli C, Zangrillo A. Reducing mortality in cardiac surgery with levosimendan: a meta-analysis of randomized controlled trials. J Cardiothorac Vasc Anesth. 2010 Feb;24(1):51-7. doi: 10.1053/j.jvca.2009.05.031; PMID: 19700350.

s60. Delaney A, Bradford C, McCaffrey J, Bagshaw SM, Lee R. Levosimendan for the treatment of acute severe heart failure: a meta-analysis of randomised controlled trials. Int J Cardiol. 2010 Feb 4;138(3):281-9. doi: 10.1016/j.ijcard.2008.08.020; PMID: 18817994.

s61. Xu ZD, He WX, Zhao YF, Xia SX, He B, Yang T, Cao DX, Peng SL, Li J, Cao MH. [Effect of levosimendan on B-type natriuretic peptide levels in patients with advanced heart failure: a meta-analysis]. Nan Fang Yi Ke Da Xue Xue Bao. 2009 Oct;29(10):2027-9; PMID: 19861257.

s62. Zangrillo A, Biondi-Zoccai G, Mizzi A, Bruno G, Bignami E, Gerli C, De Santis V, Tritapepe L, Landoni G. Levosimendan reduces cardiac troponin release after cardiac surgery: a meta-analysis of randomized controlled studies. J Cardiothorac Vasc Anesth. 2009 Aug;23(4):474-8. doi: 10.1053/j.jvca.2008.11.013; PMID: 19217315.

s63. Cleland JG, Freemantle N, Coletta AP, Clark AL. Clinical trials update from the American Heart Association: REPAIR-AMI, ASTAMI, JELIS, MEGA, REVIVE-II, SURVIVE, and PROACTIVE. Eur J Heart Fail. 2006 Jan;8(1):105-10. DOI: 10.1016/j.ejheart.2005.12.003; PMID: 16387630

s64. Cleland JG, Ghosh J, Freemantle N, Kaye GC, Nasir M, Clark AL, Coletta AP. Clinical trials update and cumulative meta-analyses from the American College of Cardiology: WATCH, SCD-HeFT, DINAMIT, CASINO, INSPIRE, STRATUS-US, RIO-Lipids and cardiac resynchronisation therapy in heart failure. Eur J Heart Fail. 2004 Jun;6(4):501-8;PMID: 15182777.
